# Measuring the value of social engagement in adults with and without autism

**DOI:** 10.1186/s13229-015-0031-2

**Published:** 2015-06-12

**Authors:** Indu Dubey, Danielle Ropar, Antonia F. de C Hamilton

**Affiliations:** School of Psychology, University of Nottingham, University Park, Nottingham, NG7 2RD UK; Institute of Cognitive Neuroscience, University College London, Alexandra House, 17 Queen Square, London, WC1N 3AR UK

**Keywords:** Social motivation, Autism, Direct gaze, Social reward, Adult behaviour, Social seeking

## Abstract

**Background:**

Differences in social communication are commonly reported in autism spectrum condition (ASC). A recent theory attributes this to a reduced motivation to engage with others, that is, deficits in social motivation. However, there are currently few simple, direct, behavioural ways to test this claim. This study uses a new behavioural measure of social motivation to test if preferences for direct gaze and face stimuli are linked to autistic traits or an ASC diagnosis. Our novel choose-a-movie (CAM) paradigm measures the effort participants invest to see particular stimuli. This aspect of social motivation is also known as social seeking.

**Methods:**

In experiment 1, 80 typical adults completed the CAM task and a measure of autistic traits. In experiment 2, 30 adults with ASC and 24 age/IQ-matched typical adults completed the CAM paradigm.

**Results:**

The results from study one showed that typical adults prefer social stimuli over non-social, but this preference is weaker in those with higher levels of autistic traits. In study two, adults with ASC showed a significant reduction in their preference for direct gaze but little difference in their preference for faces without direct gaze.

**Conclusions:**

These data show that social motivation can be measured in a simple, direct, behavioural paradigm. Furthermore, adults with ASC prefer direct gaze less than typical adults but may not avoid faces without direct gaze. This data advance our understanding of how social motivation may differ between those with and without autism.

## Background

Every day, people make choices about their social interactions—to continue work or meet with friends, to watch that cute baby video on YouTube or not. Measuring the motivational forces behind these types of choice is important in the emerging study of social motivation and will help us to understand individual differences in social preference. Here, we use a novel choose-a-movie paradigm to quantify preferences for social stimuli with and without direct gaze and demonstrate that these preferences differ in line with autism traits and autism diagnosis.

Repeated studies demonstrate that certain stimuli, including food, money and some social stimuli are valued by participants and engage ‘reward related’ brain circuits [1]. People place higher value on genuine social smiles than polite smiles and give away higher monetary rewards to see genuine smiles [2]. Another study showed that typical heterosexual males exert more effort to watch the images of attractive females than average-looking females [3]. Gaze is one social cue which seems to be particularly rewarding. Seeing an attractive face making eye contact engages brain systems linked to reward [4]. Also, infants fixate longer on stimuli with direct eye gaze compared to averted gaze [5]. However, there may also be individual differences in the value people attach to social stimuli. It was recently suggested that people with autism spectrum condition (ASC), a disorder affecting social interaction [6], may differ in their motivation to engage or affiliate with others [7–9]. In particular, differences in responsiveness to direct eye contact have been reported in ASC [10–12], which could reflect indifference or even negative arousal responses to direct gaze in this population [13, 14].

Social motivation can be measured in terms of social orientation, social seeking and social maintaining [8]. Social orientation is defined as giving attentional priority to social cues or social information. Several studies suggest that children with ASC look less towards faces and social stimuli than typical children [15, 16]. Social maintaining is described as ‘individuals’ desire to engage with others over sustained period of time’ [8]. Studies suggest that participants with ASC do not engage in reputation management [17]; do not attempt to re-engage an adult [18]; and do not flatter another person [8]. Social seeking is a concept which is typically understood as liking a stimulus (getting hedonic pleasure from it) and wanting it (making an effort to get it). The present paper focuses on this aspect of social motivation.

Previous studies of social seeking in autism have primarily used either fMRI measures or self-report. The fMRI studies suggest that there might be atypical activation in the ‘reward related’ brain systems during social interactions in ASC [19–22]. Studies using self-report measures suggest that people with ASC experience less pleasure from social contacts [23]; and do not express loneliness despite reporting lower companionship and reciprocity in their peer networks [24]. These results are consistent with the theory of reduced social motivation in ASC. One limitation of the previous methods used in these studies is that they can only be used to study a small subgroup of people with ASC who have sufficient language and insight to complete self-report measures or be able to cope with the scanning environment. This means that the generalisability of these results remain limited. A further limitation is that these studies do not distinguish between different social cues. Typical adults find direct gaze more rewarding than averted gaze [4] but those with ASC may be indifferent [10, 25].

Some researchers have used behavioural paradigms to explore ‘social seeking’ aspect of social motivation. One such paradigm was used by Hayden et al. [3] who evaluated approach behaviour for attractiveness in typical adults. They used an ‘effort task,’ in which participants were shown images of faces with different levels of attractiveness for a very brief period. The participant could press a difficult combination of keys to increase the exposure time of the image. A very similar key press paradigm has been used by Ewing et al. [26]. They measured the reward value for three categories faces, cars and inverted faces in autism. In both these studies, the critical stimuli were visible to the participant when they made a decision to view or avoid it. This means that participants could be choosing to view a particular image on the basis of any number of features, including low level differences unrelated to social cognition. One way to get around this problem is to ask participants to select a patterned box on a computer screen which is linked to the stimulus they would like to see. For instance, if a blue striped box always shows social stimuli and a green spotty one shows objects, then a person could make their choice prior to seeing the actual stimulus. As associative learning has been found to be intact in individuals with autism [27, 28], a paradigm of this kind should be a suitable way to test for social seeking without the influence of lower level cues.

**Fig. 1:**
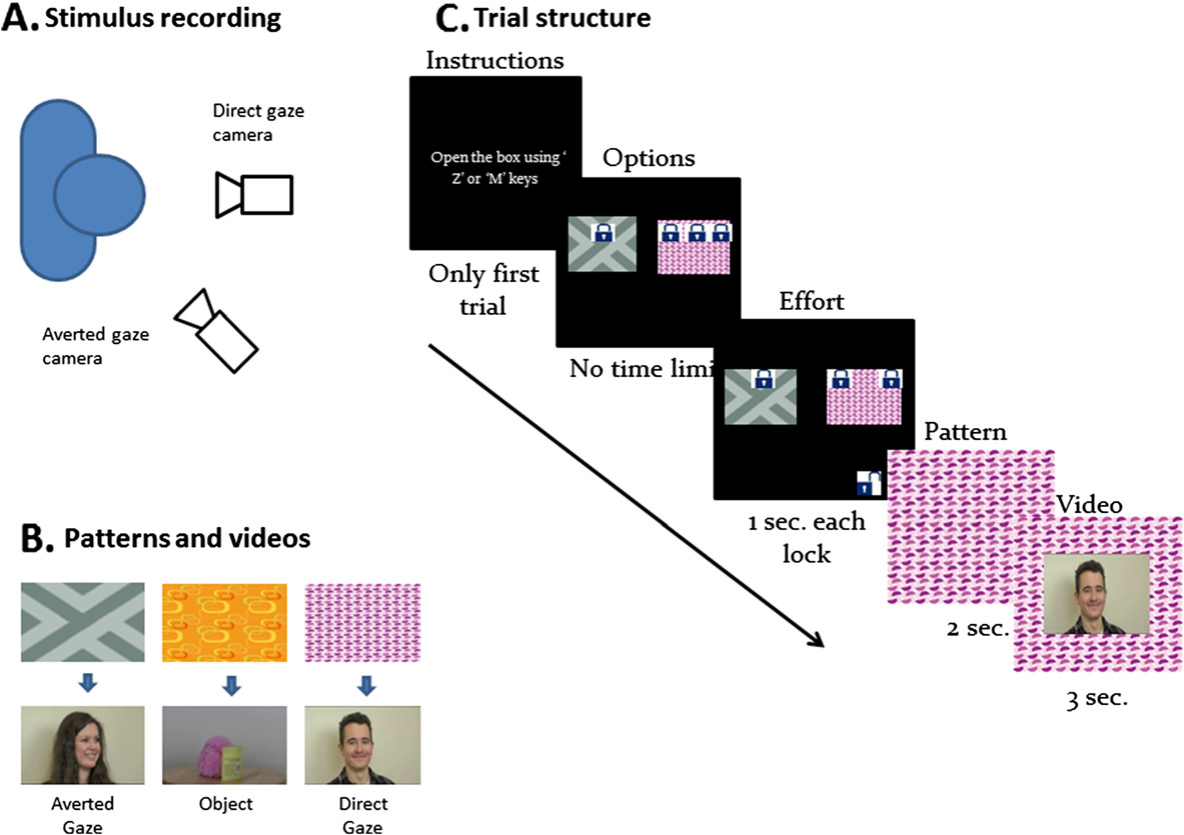
**a** Configuration for recording social videos. Two cameras simultaneously captured the actor’s direct gaze and averted gaze. **b** Stimuli and patterns. Three different patterns were linked to the three different categories of video. Different participants learnt different associations between the pattern and the video categories. **c** Trial structure. Participants first see two boxes with a variable number of locks on each. They chose which locks to remove by pressing keys. When all locks on one box are removed, the box expands to fill the screen and a video plays for 3 s

The present study uses a simple behavioural paradigm which quantifies social seeking in typical adults and adults with ASC. On each trial, the participant must choose one of two boxes to open (see Fig. 1). Their decision is influenced by knowledge of the category of video in each box (e.g. the pink stripy box always contains a video of a smiley person looking directly at the viewer) and by the number of locks present on the box (a box with three locks needs more keyhits and thus more effort to open than a box with one lock). Thus, the participant must trade-off their desire to see a particular video against the effort required. On different trials, participants make choices between direct gaze and averted gaze movies; between direct gaze and object movies; or between averted gaze and object movies. We can model performance with a multilevel logistic regression (see Methods) in which the choice made on each trial is a function of the effort needed (quantified as the difference in locks between the two boxes) and the stimulus category linked to each box. This model includes individual differences in autistic traits (measured by AQ) or autism diagnosis as a predictor. We hypothesise that participants with autism or autistic traits may have differences in social motivation. If this is the case, they will value social stimuli less and this will lead to an interaction between the stimulus category, effort and the autistic features in our model.

## Methods

### Stimuli

Three sets of movie stimuli—social direct gaze, social averted gaze and objects—were developed for the experiment. For the filming sessions, actors (five males and five females) were instructed to look up and smile in greeting as if called by a friend. Two video cameras were used to capture the same smile from straight and averted gaze directions simultaneously (for schematic presentation, see Fig. 1a. Thus, the direct gaze movies show the actor smiling directly toward the camera, while the averted gaze movies show the same smile with the actor looking away from the camera. For non-social movies, ten pairs of common household objects such as a bowl and a jar, or a paint pot and a brush were video recorded while slowly rotating on a turntable. Like the social movies, these stimuli include gradual movement causing some changes in object orientation but have no social content. The final movies were saved at 320 × 180 pixel resolution and every movie had a duration of 3 s. In addition to the video stimuli, three abstract coloured patterns were generated to use as cues to each movie category.

### Chose-a-movie task design

The choose-a-movie (CAM) task was presented using MATLAB (6.1, The MathWorks Inc. 2000) and Cogent 2000. On each trial of the CAM paradigm, participants saw two coloured boxes on the screen with between one and three locks on each box (see Fig. 1c). They could choose to open one of the boxes and would then see the movie associated with that box. Initially, participants completed 21 familiarisation trials, in order to learn how the task worked and what types of boxes and movies were present in the study. For the first 15 trials, only one box with one lock was presented at a time. For the next six trials, two boxes were presented with just one lock on each box. Participants learnt that when all locks on one box were removed, that box would open to reveal a movie. Opening each lock required one keyhit and then an animation played for 1 s showing the lock being removed. Each key press was a distinct action, and participants were not able to make quick finger movements to remove multiple locks. There were three possible boxes, each marked with a distinctive coloured pattern. There was a consistent mapping between the coloured pattern of the box and the category of movie which was shown when that box opened (Fig. 1b. The mapping was counterbalanced across participants.

Overall, participants completed 180 experimental trials. These comprised of 60 trials with a choice between direct gaze and averted gaze; 60 choices between direct gaze and object; and 60 choices between averted gaze and object. Within each set of 60 trials, 32 showed 3 locks on one box and 1 on the other; 8 showed 2 locks on one box with 1 on the other, 8 showed 3 locks on one box with 2 on the other, and 12 showed equal numbers of locks on each box. The box with the larger number of locks was pseudorandomly assigned to the left or right side of the screen with equal probability for each number of locks. On each trial, a participant could choose to open the box with fewer locks (requiring less button presses and time) or the box with more locks (requiring more button presses and time). Thus, participants were encouraged to make a trade-off between the effort required to open the box and their preference for a particular movie category.

### Experiment 1

#### Participants

Eighty adults (39 females, age 18–43 years) participated in the study. Participants were recruited by posters in different science/business/arts departments of the University of Nottingham. One participant reported having a positive family history of autism spectrum disorder in a first-degree relative but was not excluded.

#### Procedure

Ethical approval for study one was provided by the ethics committee of the School of Psychology, University of Nottingham. The study was conducted in accordance with the declaration of Helsinki. Participants first gave written informed consent to take part in the study. They then completed the “Adult Autism Spectrum Quotient” (AQ) [29]. Two of our participants scored above the cut-off (32) on AQ for the general population [29] but had never been assessed for or diagnosed with an ASD. Next, the CAM task was presented using MATLAB with Cogent toolbox.

#### Data analyses

Each participant’s data from the 180 multi-lock choice trials was split into three sets of 60 trials, one set for direct-gaze vs. object choices; one for averted gaze vs. object; one for direct gaze vs. averted gaze. We fitted three separate models for the three different choice pairs, so model 1 includes only trials where participants chose between direct gaze and objects; model 2 includes only trials where participants chose between averted gaze and objects; model 3 includes only trials where participants chose between direct and averted gaze. For each model, we fitted data from all participants simultaneously using a mixed-level generalised linear model in SPSS, i.e. data for all 80 participants for the direct-gaze vs. averted gaze choice pair was entered into a single, mixed-level model. This expands on the approach of Shore and Heerey [2].

Our model used the logistic link function

$$ p\left(\mathrm{left}\right) = {e}^t/\ \left(1 + {e}^t\right) $$

in which *p (left)* is the probability of selecting the box on left and *t* is the difference in the utility between the two boxes. Utility was modelled as a linear function of the effort required to open the box, the stimuli type and their interaction.

$$ t = {\beta}_1{x}_1+{\beta}_2{x}_2+{\beta}_3{x}_3 $$

Here, *x*_1_ is the difference in the number of locks on the two boxes (−2 to 2), *x*_2_ is a dummy variable coding the identity of the item in the left box (e.g. 1 = direct gaze; −1 = averted gaze) and *x*_3_ is the interaction between *x*_1_ and *x*_2_. In this model, we predicted whether the participant would choose the item on the left based on the following factors: *effort -* the relative number of locks on the left (−2, −1, 0, 1, 2) and *stimulus -* the identity of the stimulus on the left (e.g. direct gaze/averted gaze). Other predictors were included as participant-level factors, including *AQ -* the participant’s AQ score, their age and gender. Thus, a single mixed-level model was used to analyse all the data. We tested for main effects of all predictors and also for interactions of effort by stimuli, effort-by-AQ, stimuli-by-AQ; and effort-by-stimuli-by-AQ. Thus, it was a mixed-level model (generalised linear regression) using a logistic link function with participant ID as a between-subjects factor. Data were analysed in SPSS, and results are reported in terms of the Wald statistic provided.

### Experiment 2

#### Participants

Thirty adults with ASD (nine females) and 24 matched typical adults (ten females) between age range of 20–60 years participated in the study. Participants were recruited from the autism/participant database of the Institute of Cognitive Neuroscience, University College London.

#### Procedure

Ethical approval for study two was provided by the University College London graduate school ethics committee. The study was conducted in accordance with the declaration of Helsinki. All the participants gave written informed consent to take part in the study. All the participants were tested for their intellectual abilities. The verbal IQ (ASD = 117.83 ± 14.71, controls = 117.88 ± 15.80), performance IQ (ASD = 110.17 ± 14.66, controls = 114.88 ± 13.42) and full-scale IQ (ASD = 115.83 ± 13.87, controls = 118.04 ± 12.79) was measured by Wechsler Adult Intelligence Scale (WAIS) [30]. The groups did not differ in their gender ratio (χ^2^ (1, *N* = 54) = 0.796, *p* = 0.40). The participants in the ASD group all had an independent clinical diagnosis of ASD. The diagnosis was confirmed by documentary proof provided by each participant. As a current measure of severity of symptoms all except one participant were evaluated on Autism Diagnostic Observation Schedule (ADOS). The ADOS scores showed that 12 participants qualified for the category of ‘autism’ , 12 for the category of ‘autism spectrum disorders’ , while five participants had low score thus not qualifying for either ‘autism’ or ‘autism spectrum disorder’ category on ADOS. We did not have ADOS evaluation for one participant. Irrespective of the ADOS scores all the participants in the ASD group had a clear diagnostic history and clinical diagnosis of autism spectrum condition from independent clinicians. As an additional measure of autism severity we administered the ‘Adult Autism Spectrum Quotient ’ (AQ) [29] to all the participants and found that the two groups were significantly different (*p* < 0.0001) on their mean AQ score (ASD = 35.25 ± 9.23, controls = 20.33 ± 8.69). All the participants then completed the CAM paradigm in the lab setting.

#### Data analyses

Data was analysed in the same way as described above for experiment 1. This was a mixed-level model (generalised linear regression) using a logistic link function with participant ID as a between-subjects factor, and age and gender as covariates. We tested for main effects of effort, stimuli, and group (ASD and matched typical) and also for interactions between these factors. Data was analysed in SPSS and results are reported in terms of the Wald statistic provided. Note that the influence of the videos on choice behaviour could be reflected in either an effect of stimulus or in a stimulus by effort interaction (if effort differences induce ceiling effects), and we can consider either as evidence for motivation towards a particular type of video.

## Results

**Fig. 2:**
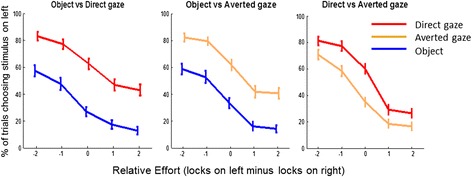
Choices according to effort and stimuli. Each plot shows how often (%) the participant chose the left box for a particular level of effort. The *coloured lines* indicate which stimulus category was in the left box on each trial. For example, in the *left-hand plot*, the *red line* above the *blue line* indicates participants preferred direct gaze videos

In experiment 1, we tested 80 typical adults using the choose-a-movie paradigm and measured their autistic traits using the ‘Adult Autism Quotient Scale’ (AQ) [29]. Results (see also Fig. 2) showed choices in the direct gaze vs. object trials were reliably influenced by the stimulus category (Wald chi square = 17.40, *p* < .001), effort (Wald chi square = 17.04, *p* = .002) and autistic traits (Wald chi square = 3.88, *p* = .049). Critically, there was a significant interaction between stimuli and AQ (Wald chi square = 6.03, *p* = .014). No other interaction between these factors was significant. Also, there was no significant effect of age or gender on the choice of the participants. For choices between averted gaze movies and objects, participants’ choices were again significantly influenced by interaction between stimuli and AQ (Wald chi square = 8.99, *p* = .003) (other effects were similar to before, see Table 1). For choices between averted gaze movies and direct gaze movies, the interaction between stimuli and AQ was a marginal predictor of choice (Wald chi square = 3.51, *p* = .061). Overall, these results show that within the typical population, the preference for a social (direct or averted gaze) movie over an object movie is linked to autistic traits. Participants with higher levels of autistic traits show a weaker social preference, indicating that they attach less value to seeing a social movie, however between two social stimuli participants’ choice is marginally influenced by the gaze direction in the stimuli.

**Table 1:** Logistic regression for experiment 1: factors influencing participants’ decision to choose stimuli on the left

	Object vs. direct gaze	Object vs. averted gaze	Direct vs. averted gaze
	(Wald χ^2^, *p*)	(Wald χ^2^, *p*)	(Wald χ^2^, *p*)
Stimulus	17.41, *p* < 0.0001	20.02, *p* < 0.0001	9.72, *p* = 0.002
Effort	17.04, *p* < 0.002	20.51, *p* < 0.0001	18.60, *p* = 0.001
AQ	3.88, *p* = 0.049	0.628, *p* = 0.428	0.019, *p* = 0.889
Stimulus by AQ	6.03, *p* = 0.014	8.995, *p* = 0.003	3.51, *p* = 0.061
Stimulus by effort	3.41, *p* = 0.492	3.25, *p* = 0.517	2.45, *p* = 0.654
Effort by AQ	4.46, *p* = 0.348	7.61, *p* = 0.107	6.46, *p* = 0.167
Stimulus by effort by AQ	2.81, *p* = 0.591	4.50, *p* = 0.343	2.03, *p* = 0.730
Age	0.143, *p* = 0.705	0.581, *p* = 0.446	0.130, *p* = 0.719
Gender	0.510, *p* = 0.475	2.40, *p* = 0.121	1.35, *p* = 0.246

**Fig. 3:**
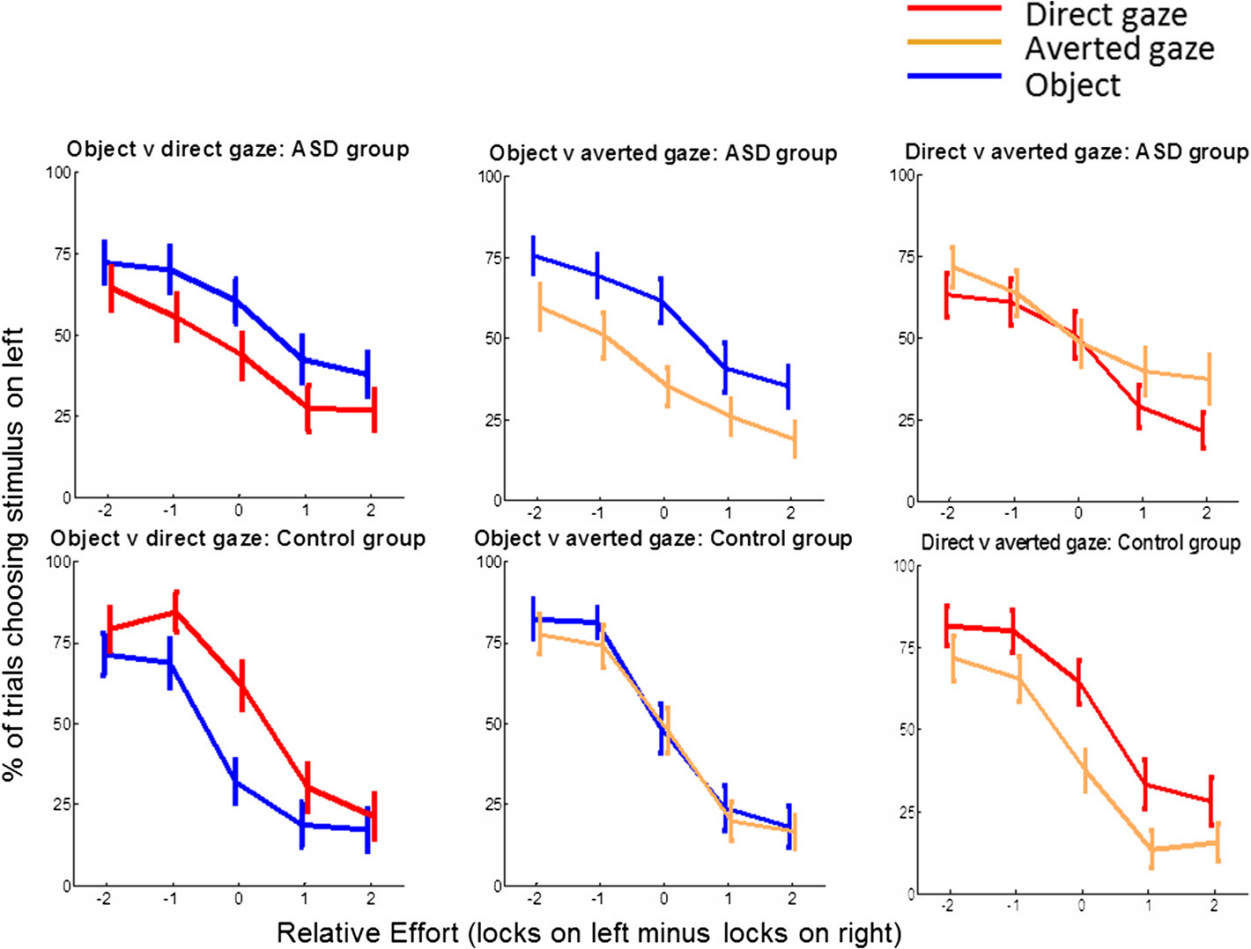
Choices in participants with (*top row*) and without (*bottom row*) autism. As in Fig. 2, the *x axis* represents effort and the *lines* show how often participants chose to view the movie on the left, for direct gaze (*red*), averted gaze (*orange*) and object (*blue*) movies

In experiment 2, we tested 30 able adults with a clinical diagnosis of ASC and 24 typical adults matched for age and IQ, using the same task and analysis. Results are shown in Fig. 3. Reliable effects of the group are reported here, and all effects are given in Table 2. To further understand the group interaction results, we conducted the same analyses in the typical and ASC groups separately. These results are presented in Table 3.

**Table 2:** Logistic regression for experiment 2: factors influencing participants’ decision to choose stimuli on the left

	Object vs. direct gaze	Object vs. averted gaze	Direct vs. averted gaze
	(Wald χ2)	(Wald χ2)	(Wald χ2)
Stimulus	0.022, *p* = 0.883	2.96, *p* = 0.086	0.65, *p* = 0.422
Effort	49.51, *p* < 0.0001	53.91, *p* < 0.0001	44.25, *p* < 0.0001
Group	0.354, *p* = 0.552	0.700, *p* = 0.403	0.014, *p* = 0.906
Stimulus × group	3.10, *p* = 0.083	0.99, *p* = 0.320	3.22, *p* = 0.073
Stimulus × effort	4.05, *p* = 0.400	0.45, *p* = 0.978	10.76, *p* = 0.029
Effort × group	10.65, *p* = 0.031	8.58, *p* = 0.073	4.25, *p* = 0.373
Stimulus × effort × group	11.99, *p* = 0.017	2.26, *p* = 0.689	9.43, *p* = 0.051
Age	1.65, *p* = 0.199	1.26, *p* = 0.261	1.45, *p* = 0.228
Full-scale IQ	0.140, *p* = 0.709	0.011, *p* = 0.918	0.212, *p* = 0.645

**Table 3:** Logistic regression by group for experiment 2

	Object vs. direct gaze		Object vs. averted gaze		Direct vs. averted gaze	
	(Wald χ2)		(Wald χ2)		(Wald χ2)	
	Control	ASD	Control	ASD	Control	ASD
Stimulus	1.417 *p* = 0.234	1.670 *p* = 0.196	0.228 *p* = 0.633	4.346 *p* = 0.037	2.672 *p* = 0.102	0.664 *p* = 0.415
Effort	33.398 *p* < 0.0001	20.705 *p* < 0.0001	42.311 *p* < 0.0001	27.363 *p* < 0.0001	30.603 *p* < 0.0001	16.035 *p* = 0.003
Stimulus × effort	13.702 *p* = 0.008	1.889 *p* = 0.756	1.125 *p* = 0.890	2.522 *p* = 0.641	11.833 *p* = 0.019	11.259 *p* = 0.024
Age	0.181 *p* = 0.671	4.038 *p* = 0.044	0.761 *p* = 0.383	3.898 *p* = 0.048	0.010 0.922	3.179 *p* = 0.075
Full-scale IQ	0.846 *p* = 0.358	1.795 *p* = 0.180	3.351 *p* = 0.067	1.372 *p* = 0.241	9.976 *p* = 0.002	1.612 *p* = 0.204

In the choice between direct gaze movies and objects, there was a three-way interaction between effort, stimuli and diagnostic group (Wald chi square = 11.99, *p* = .017) and interaction of effort by group (Wald chi square = 10.65, *p* = .031). This can be seen in Fig. 3 as direct gaze videos are more often chosen in the typical group (red line higher than blue line) but are less often chosen in the autism group (blue line higher). The analysis of separate groups suggests this effect is driven by a stimulus by effort interaction in the typical sample (Wald chi square = 13.7, *p* = 0.008) which was not present in the ASC sample. In the choice between averted gaze movies and objects, there were no reliable effects of group. In the choice between direct and averted gaze movies, there was a marginal interaction between effort, stimuli and group (Wald chi square = 9.43, *p* = .051). In the analysis of separate groups, there was a stimulus by effort interaction for both the typical (Wald chi square = 11.8, *p* = 0.019) and ASC groups (Wald chi square = 11.3, *p* = 0.024).

## Discussion

Using our novel behavioural choose-a-movie task, we show that typical adults have a reliable preference for social stimuli, and that this preference is reduced in those with more autistic traits. Further testing of participants with a diagnosis of ASC shows that these participants have a reduced preference for direct gaze. Together, these data suggest that typical adults value direct gaze more than objects or averted gaze, whereas adults with ASC do not show this preference. We discuss these results in terms of measuring reduced social motivation in autism.

Our novel choose-a-movie paradigm provides a straightforward way to quantify social motivation in individual participants. It differs from previous measures of social motivation in several ways. Eye tracking measures of social orienting [31, 32] have been widely used, but it is hard to determine if results are driven by low-level stimulus features or even by differences in the oculomotor system [33]. In our study, when the participant makes a choice, they see only abstract cues (boxes) on the screen and must be guided by an internal value signal. This is similar to Shore and Heerey’s [2] study with typically developing individuals, but we go further and use video stimuli which have higher ecological validity than photos. Neuroimaging measures of stimuli’s value have also been used [19, 21, 22] but are hard to apply across a wide range of participants. Using the CAM task, we are able to show that typical adults value a smiling face with direct eye contact over a smiling face with averted gaze or objects and value an averted gaze face over objects. Participants are prepared to put in more effort to see their preferred stimulus.

We then used the CAM task to test the hypothesis that participants with ASC differ from typical adults in social motivation [8]. The CAM task targets the social seeking aspect of social motivation that has not been tested in prior behavioural studies. CAM allows us to evaluate the influence of stimuli, effort and their interaction along with other variables on the choice behaviour of the participants. Our results suggest that in relation to their autistic traits, participants are differentially influenced by the stimuli but not by the effort. However, if participants are diagnosed with ASD, they are influenced differently by both stimuli and effort. There are two possible interpretations of this result. One is that autistic traits (as measured by AQ) might not impact on behaviour in quite the same way as a true autism diagnosis. The second is that a stimulus by effort interaction may arise when participants are strongly driven by effort and show floor/ceiling effects for the −2/+2 lock conditions, meaning that stimulus effects are only visible in the intermediate conditions. Visual inspection of the plots suggests the latter. Participants in experiment 2 completed the study as part of a day-long visit to the lab with many other studies, so it is plausible to suggest they were heavily influenced by effort. Because of this effect, we interpret both a main effect of stimulus and a stimulus by effort interaction in the same way, but further study will be required to determine if there are also subtle differences between those with high autistic traits compared to those with a diagnosis of autism. In the future, the levels of effort can also be increased to present a more extreme effort comparison such as one vs. ten locks, to allow a stronger effort effect.

Our data show that typical participants value direct gaze more than objects while participants with autism do not. It is unlikely that the repetitive nature of stimuli might influence non-social preference in ASC, as all three sets of stimuli had an equal number of different movies (ten social and ten objects). Furthermore, the object movies showed a single slow rotation ensuring that the object videos are not more repetitive than the social videos. The above reported finding could be due to a general reduction in social motivation in autism or to a more specific indifference towards direct gaze. Preference for non-social over social stimuli is also reported by Chevallier et al. [34], however they found that within social stimuli people with ASD might prefer direct gaze stimuli more. In contrast, the results of the direct–averted gaze comparisons in our study show a marginal interaction with group and we find that both groups show stimulus by effort interactions in opposite directions. This implies that typical and ASC participants value direct and averted gaze differently and supports the primary result that participants with autism do not value social stimuli with direct gaze as much as typical adults. Similar findings have been reported earlier emphasising the significance of communication intent [10] or approach motivation [12] through direct eye contact in typical people and lack of it in ASC.

However, our data do not show extreme aversion to direct gaze in ASC. Participants with ASC would sometimes choose to look at the direct gaze stimuli if it required less effort than the other option. This implies that the lack of approach towards direct eye-gaze stimuli in ASC might be driven more by lack of interest in social interaction than by aversion from the eyes [11]. Further to the group analysis, CAM allows us to calculate a simple metric for each individual as the percentage of trials where that person chose the social option (averaging over all effort levels, which are balanced across all options). These individual metrics might be helpful in understanding the strength of social/non-social preference in individual participants and might point towards the factors influencing their choice behaviour. However, we would not yet recommend this for clinical use.

The finding that social direct gaze is valued less in participants with ASC is compatible with a number of previous reports. Failure to make or respond to eye contact is a diagnostic indicator for ASC [6], and studies suggest eye contact is hyper-arousing for children with ASC [14]. We note also that the previous studies which have measured social reward in ASC [19, 21, 22] have used face stimuli with direct gaze, and so findings from those studies are compatible with the current data showing differences in the valuation of direct gaze stimuli.

When given the opportunity to view movies without direct gaze, the participants with autism in the present study did not differ from the typical participants. However, we note that the typical participants in experiment 2 did not show the same preference for averted gaze movies as those in experiment 1—this could be due to differences in the age, IQ or demographics between studies or to the smaller sample size in experiment 2. Further investigation of how both typical participants and those with ASC value social stimuli that do not involve direct gaze would be valuable. In particular, it would be useful to test the breadth of differences in social motivation—do participants with autism avoid all social stimuli or only those that directly signal engagement?

## Conclusions

Overall, our findings suggest that the value of social stimuli can be measured using a simple behavioural method, which controls for lower level visual features of the stimuli and gives a precise measure of social seeking. We demonstrate the clinical importance of this approach by quantifying how people with ASC value videos of direct gaze less than typical participants. This could be due to a general difference in social motivation, or it could be due to a more specific difference in the value of direct gaze itself. Further investigation of these two possibilities will be needed. In the future, the CAM paradigm might be a helpful tool for estimating the expected efficacy of social reward-based behavioural intervention used for developmental or psychiatric disorders.

## Abbreviations

AQ: the adult autism spectrum quotient
ASC: autism spectrum condition
CAM: choose-a-movie
